# Why is Turkey losing its doctors? A cross-sectional study on the primary complaints of Turkish doctors

**DOI:** 10.1016/j.heliyon.2023.e19882

**Published:** 2023-09-10

**Authors:** Salim Yılmaz, Seher Koyuncu Aydın

**Affiliations:** a Istanbul Arel University, Faculty of Health Sciences, Assistant Professor at Health Management Department, Istanbul, Turkiye; b Sancaktepe Sehit Prof.Dr. Ilhan Varank Training and Research Hospital, Research Assistant at Gynecology and Obstetrics, Istanbul, Turkiye

**Keywords:** Doctors, Doctor migration, Doctor complaints, Primary complaints, Turkey

## Abstract

In 2022, Turkey encountered the formidable task of addressing an unprecedented loss of medical doctors and seeking remedies for potential issues within the healthcare system. This study set out to explore the inclination of 402 actively practicing Turkish doctors to depart from Turkey, assess the socio-demographic and socio-economic factors influencing this trend, and establish the hierarchy of raised concerns among doctors. Employing a cross-sectional and analytical approach, the study drew comparisons between doctors' demographic characteristics and the significance of their grievances, while also examining the correlation between the importance of complaints and the desire to remain in Turkey. The doctors' primary complaints encompassed financial challenges, instances of violence in the healthcare sector, and insufficient examination durations. The migration of doctors poses a substantial risk to healthcare accessibility, public health, and the sustainability of Turkey's healthcare delivery capacity. To mitigate this risk and curb doctor migration, corrective measures must be implemented to improve working conditions. Additionally, there is a need for further scientific research focusing on doctors' concerns, particularly in developing countries like Turkey, to expand the current body of literature on this subject.

## 1 Introduction

The demands and challenges encountered by physicians are numerous and diverse, exerting significant impacts on their well-being, satisfaction, and ability to deliver top-quality care to patients. Furthermore, the training of physicians is a lengthy and costly process, and the loss of these invaluable healthcare professionals can entail substantial financial risks and jeopardize public health if the issues of working conditions and well-being are not adequately addressed [1,2]. Grievances like financial insufficiency, prolonged patient waiting times, shift work, malpractice concerns, and mobbing are all too prevalent among healthcare professionals worldwide [3]. Despite the profound impact of doctor migration on healthcare systems worldwide, the number of studies conducted in an academic sense, aimed at understanding the reasons behind it and developing policy solutions based on these reasons, remains insufficient. However, the loss of doctors from countries already struggling with healthcare service delivery weakens the healthcare system, leading to prolonged patient waiting times and increased risks in terms of healthcare service costs [4]. Therefore, in order to ensure the well-being of doctors and alleviate the risks associated with their migration, it is necessary to identify and address the concerns raised by doctors through comprehensive strategies.

In recent years, Turkey has become a prominent example of physician migration, with a rising trend among doctors that potentially exposes significant risks to the delivery of healthcare services within the country [5]. Understanding the context of physician migration and deciphering the underlying reasons that drive doctors to migrate are crucial first steps towards mitigating its impacts on Turkey's healthcare system. Moreover, an examination of Turkey's physician migration scenario provides an opportunity to draw insights from similar contexts in other countries, thus identifying successful strategies and approaches to manage this phenomenon. By doing so, it may be possible to enhance the quality and accessibility of healthcare services while improving physician welfare and job satisfaction [6]. Therefore, investigating the case of physician migration in Turkey carries substantial importance. It holds the potential to provide valuable knowledge and strategies that could improve not only the sustainability of local healthcare services but also contribute positively to the broader landscape of global healthcare services. In this study, our objective is to assess the prevalence and repercussions of these issues on physicians while exploring potential remedies and recommendations to tackle them. Additionally, we will delve into other common complaints such as organizational difficulties, violence, apprehensions about the future, constraints related to compulsory service and assignments, physical conditions, discrediting, and regulatory matters, all of which contribute to the overall burden faced by physicians. Within the context of Turkey, our study will examine the significance of physician complaints in relation to various sociodemographic characteristics and their intentions to leave the country. By comprehending the root causes and consequences of these complaints, we aspire to illuminate the challenges experienced by physicians and provide valuable insights to enhance working conditions and overall well-being within this vital profession.

## 2 Background

Considering factors such as financial challenges, extended work hours, heightened error risk, and the substantial cost of mistakes, comprehending the level of pressure experienced by doctors is crucial. Despite enduring a lengthy and demanding education, they continue to grapple with these issues throughout their professional lives [7]. The demanding workload induces fatigue, amplifying the likelihood of malpractice incidents, and doctors must fulfill their duties while harboring concerns about potential errors. Additionally, the inadequate remuneration compared to the anticipated income, spanning from medical school education to their careers, presents a notable predicament. Thus, as highlighted in Yang et al., [8] study, monetary rewards beyond base salaries play a significant role in motivating doctors. Furthermore, Decker [9] conducted a comparison of Medicaid physician payments across various U.S. states and discovered that higher Medicaid fees contribute to an increased presence of private doctors, particularly in medical and surgical specialties. This research also noted that higher fees contribute to longer doctor-patient interactions, thereby enhancing healthcare access quality. Lin and colleagues [10] examined the non-linear escalation of working hours among healthcare professionals and reported that burnout rates increased at a rate one and a half times faster after reaching 60, 74, and 84 h per week. Moreover, Soh and colleagues [11] identified a correlation between malpractice incidents and prolonged work hours in their studies. Hanganu and colleagues [12] emphasized that night shifts similarly elevate stress, fatigue, and risk, similar to extended work hours.

It is widely acknowledged that factors like salary, working hours, and shift work hold immense importance in determining job satisfaction for all workers. In the healthcare sector, however, healthcare professionals have the potential to derive high levels of internal satisfaction due to intrinsic rewards such as societal respect and the fulfillment derived from positively impacting lives [13]. Among healthcare professionals, doctors hold a particularly critical position as decision-makers and the initial point of contact for patients [14]. Nevertheless, the changing world, marked by increased mass communication, has raised concerns about the erosion of respect for doctors compared to the past [15]. Factors contributing to this issue include doctor ratings on social media, the shrinking global community, social media usage, and country policies [16].

Healthcare challenges extend beyond individual concerns. Hospitals with a matrix organizational structure, for instance, often encounter problems and conflicts in their daily operational activities [17]. Hence, adjusting legislation and organizational structures within hospitals, especially in the public sector, becomes essential. Inadequacies such as a lack of management authority, inconsistency, difficulty in comprehension, frequent changes in legislation that impede compliance, and a lack of transparency can give rise to problems [18]. Moreover, legislation and regulations related to digitalization and technology may be insufficient and outdated. Topics such as telemedicine, artificial intelligence, transplantation, and euthanasia have sparked legal debates in numerous countries [[19], [20], [21]].

Organizational conflicts, particularly mobbing, can significantly impact health. A qualitative study in France revealed that doctors face reduced freedom, limited perspective, diminished passion, a decreased emphasis on care, and high levels of organizational conflict and pressure in their work environments [22]. Similarly, a study in Serbia reported that 30.4% of emergency room doctors experienced mobbing. Research indicates that conflicts and mobbing can greatly affect doctors' job satisfaction, leading to burnout and depression, particularly among young doctors [23].

In addition to personal and organizational issues, inadequate hospital resources that affect access to healthcare can have a detrimental impact on doctors' work [13]. Insufficient physical resources in healthcare facilities can complicate patient treatments, reduce doctors' motivation, and increase health and safety risks for healthcare workers [18]. When the quality of care diminishes due to hospital limitations, it poses a risk to public health and increases the potential for conflicts between patients, families, and doctors [13,18].

Due to their pivotal roles in diagnosing and treating patients, doctors maintain constant contact with patients and their families. However, tensions can sometimes arise between patients and doctors due to reasons such as the absence of a cure for certain health conditions, irreversible outcomes, and emotional distress [24]. Many countries, particularly those outside developed nations, frequently witness incidents of violence within the healthcare system, exposing doctors to psychological and physical harm [25]. Emotional and physical stress can jeopardize the safety of doctors, increase the risk of compromised performance, and lead to psychological issues such as depression, anxiety, and sleep disorders [26]. Despite the passage of time, rates of violence against doctors, which can result in injuries and fatalities, remain unchanged [27].

The number of doctors per 1000 people serves as a crucial indicator of the healthcare system's quality due to the high demand for doctors worldwide [28]. Doctors are selected from among accomplished individuals in nearly every country and undergo a lengthy and rigorous training process due to the critical nature of their roles in healthcare [29]. However, factors like rapid population growth and aging contribute to an increasing need for doctors each day to safeguard and improve community health [30]. The COVID-19 pandemic has further highlighted the demand for healthcare workers, particularly doctors [31].

Excluding the pandemic, the chronic need for doctors is evident in various countries, particularly in rural areas where accessing healthcare services is challenging, resulting in numerous issues and prompting countries to implement diverse measures. Addressing this need involves not only incentive policies, such as fees but also mandatory policies [32]. In Turkey, for instance, doctors are assigned to underserved regions after their training as part of their “compulsory service.” Once they complete their specialization, they are reassigned to obtain their specialty certificates, which are granted upon fulfilling the compulsory service requirement. Public service resignations are limited to three instances, based on the inability to work for the public again, and appointments consider the doctor-to-population ratio in specific geographic regions [33].

The scarcity of doctors puts working medical professionals in a challenging position, compelling them to keep consultation times brief, consequently increasing the risk of medical errors [34]. Research by Alqahtani et al. [35] revealed that due to time pressure, doctors achieved significantly lower diagnostic accuracy scores. Similarly, Linzer et al. [36] found that doctors' intention to leave their work environment was correlated with time constraints and job satisfaction.

Amidst these circumstances, doctor migration poses a significant threat to countries' healthcare systems. Doctor migration refers to the departure of physicians from their home countries due to economic, social, or political reasons, whether voluntary or forced [37]. While host countries benefit from bridging the doctor gap and improving healthcare service quality, sending countries face notable challenges, including increased training costs in the healthcare sector, disruption of healthcare workforce planning, diminished service quality, and difficulties in accessing healthcare services [38]. Recently, Turkey has experienced an economic crisis, financial insufficiency, heightened violence, the impact of the COVID-19 pandemic, and elevated expectations due to globalization. Collectively, these factors have negatively affected the healthcare system and contributed to an increase in doctor migration.

The objective of this study is to prioritize the primary complaints of medical doctors based on their significance, identify determinants based on doctors' characteristics, examine these determinants in relation to their desire to leave Turkey and provide insights into the expectations of medical doctors in similar countries from a sociodemographic and socio-economic perspective.

## 3 Materials and methods

### 3.1 Research type

The type of research conducted in this study can be characterized as a cross-sectional and analytical study. The study was planned with the aim of ranking various complaints and concerns of doctors at the time of the study, according to their perceived importance.

### 3.2 Research questions

In this study, our primary objective was to address the following inquiries.

• What are the primary grievances and worries of physicians employed in public hospitals in Turkey?
• How do these grievances and concerns rank in terms of perceived importance by the physicians themselves?
• Are there any variations in the ranking of these grievances and concerns based on variables such as sex, age, marital status, professional experience, and qualification?
• What is the extent of the physicians' inclination to pursue their careers as doctors in Turkey?
• Is there a correlation between the ranked grievances and the desire to continue practicing as a doctor in Turkey?

### 3.3 Time and place of the research

The data was collected from nine different public hospitals (Istanbul Sancaktepe Martyr Prof. Dr. İlhan Varank Training and Research Hospital, Istanbul Prof. Dr. Cemil Taşcıoğlu City Hospital, Eskişehir Osmangazi University Health Practice and Research Hospital, Ankara Gazi University Faculty of Medicine Hospital, İnönü University Turgut Özal Medical Center Training and Research Hospital, Muğla Yatağan State Hospital, Izmir Tepecik Training and Research Hospital, Dalaman State Hospital, Kayseri State Hospital) located in seven different cities (Istanbul, Ankara, Muğla, Izmir, Eskişehir, Malatya, Kayseri) in Turkey. The data collection period was between September 1, 2022, and November 20, 2022.

### 3.4 Universe and sample of the research

The latest health statistics yearbook shared by the Ministry of Health of Turkey indicates that it pertains to the year 2021 and states that there are 183,569 physicians [39]. When considering Turkey as the population, a minimum of 384 participants is required to be included. However, with a cross-sectional and restrictive approach, the study assumes the population to be the 9 hospitals in question. Accordingly, the population of the study consists of 7360 medical doctors who are known to work at the mentioned hospitals. To ensure reliable results, a minimum of 366 valid responses were required, considering a 5% risk of type 1 error and p and q values of 0.5. The Sample Size Calculator 3.0.1.4 program was utilized to calculate the required sample size using the formula [40]:

[40] $$n=\frac{N*p*q*t_{\alpha ,df}^{2}}{\left(N-1\right)*e^{2}}$$

After excluding questionnaires with unanswered items, a final sample of 402 surveys remained for analysis. Accordingly, it is assumed that the number of individuals reached in the study represents the population.

### 3.5 Data collection tools

The questionnaire for the study comprises three sections. The initial section encompasses inquiries about participant characteristics, including sex, age, marital status, years of medical practice, and current degree status.

The second section includes a component where physicians are requested to rank 12 complaint items in order of importance, from the most important (ranked as complaint number 1) to the least important (ranked as complaint number 12). These 12 complaints were determined based on a preliminary study conducted, which involved interviews with 8 doctors (3 specialists, 5 resident physicians). The doctors were asked to freely express their complaints, and these were subsequently categorized into 12 distinct headings without any reduction. To establish the validity of the scope, the compiled list was shared with the doctors, who provided their feedback using the Davis [41] and Lawshe [42] techniques, where they rated the appropriateness of each item as either “appropriate,” “can be improved,” or “not appropriate.” The validity rate of the scope was then calculated using the expert count index (>0.78) [38], confirming its suitability. The 12 complaints are coded and listed below for analysis, along with their respective explanations.

A. *Financial limitations*: Insufficient wages, insufficient compensation for work.
B. *Examination time*: Short examination time due to a large number of patients.
C. *Length of work hours*: Long weekly working hours per day.
D. *Shift work*: Work schedule that constantly changes due to night and day shifts.
E. *Fear of malpractice:* Fear of causing harm to the patient morally and legally due to incorrect decisions or procedures.
F. *Mobbing and organizational problems:* Pressure, bullying, or organizational problems.
G. *Violence:* Mostly psychological or physical violence from patients or their relatives, or fear of such violence occurring.
H. *Concerns about the future:* Concern or disappointment about future in the profession, economics due to political reasons, or various expectations related to the profession.
I. *Mandatory service and appointment barriers:* Mandatory appointments after graduation from medical school. The requirement to serve approximately 2 years in a geographical area for the purpose of obtaining a specialist title after specialization, and difficulties with appointments due to the characteristics of the region.
J. *Physical conditions:* Diagnosis, examination, device, equipment, building, and room inadequacies.
K. *Discrediting:* Statusal devaluation towards medicine in the country, degradation of the status and reputation of doctors, and negative public opinion.
L. *Problems with legislation and regulations:* Issues with laws and regulations related to the profession, obstacles in the application of laws and regulations, and the need for improvements in these areas.

The questionnaire used in this study is not in the form of a scale. It consists of responses to a questionnaire based on statements provided by 8 doctors and piloted with the first 30 doctors. Therefore, each question is evaluated individually, and it is not designed to assign scores or aggregate variables for hypothesis testing.

In the third part of the research, participants were asked to rate their desire to continue practicing medicine in Turkey using a Visual Analogue Scale (VAS) ranging from 0 to 10 (0: I have no desire at all; 10: I have a strong desire) [43]. The entire questionnaire was developed in Turkish by the researchers, and the participants are also native Turkish-speaking physicians.

### 3.6 Data collection

Our data was collected using a multi-stage cluster sampling technique. This method involves organizing subgroups within larger groups in a hierarchical manner. When selecting the clusters, specific criteria were considered for the hospitals. In the first stage, hospitals were clustered based on seven different cities in Turkey: Istanbul, Ankara, Muğla, Izmir, Eskişehir, Malatya, and Kayseri. Each city formed a primary cluster comprising hospitals with distinct geographical and demographic characteristics.

In the second stage, within each city cluster, hospitals were further grouped into sub-clusters based on more specific criteria. These criteria included factors such as the number of doctors in the hospitals' services, the patient demographics they catered to, and other relevant characteristics. Additionally, in two cities, one hospital from a rural area and one from an urban center were included. This brought the total number of hospitals to nine.

The data collection parts of research studies based on questionnaires in hospitals affiliated with the Ministry are carried out through the hospital's training nurses. Training nurses are responsible for distributing, collecting, and delivering the questionnaires to the researchers.

The selection of hospitals in the multi-stage clustering process was conducted with some randomness to encompass hospitals that fulfilled the established criteria and to ensure diversity in the dataset. This approach aimed to enhance the likelihood of representing various types of hospitals in Turkey, including university hospitals, state hospitals, and training and research hospitals, throughout the sampling procedure. It was acknowledged that all clusters contributed to capturing different facets of the overall population.

### 3.7 Analysis of data

**Fig. 1:**
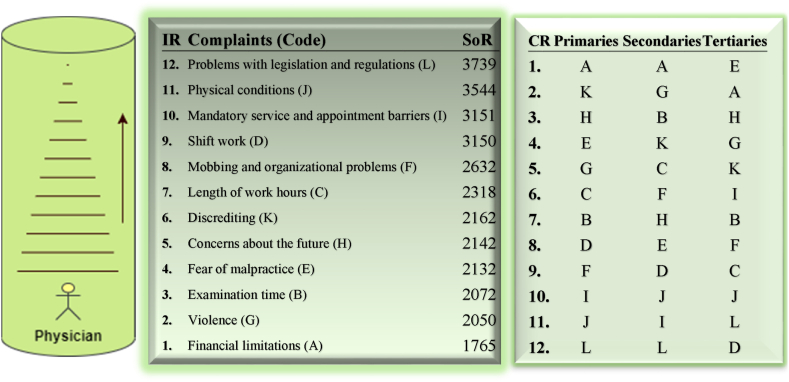
The IR of doctors' complaints and categorization using 3-point priority card.

Three data analysis methods were employed. Firstly, a matrix illustrates the complaints ranked by participants from most to least important (1–12). It shows the number of physicians who assigned each complaint a specific rank (Table 1). Secondly, a Sum of Ranks (SoR) model was established. In this model, the importance rank of each complaint is multiplied by the number of physicians who ranked it accordingly. Complaints ranked first receive rank scores equal to the number of physicians who placed them there, while those ranked twelfth receive a score obtained by multiplying by 12. The rank scores are then summed vertically to obtain the SoR scores for each complaint based on the number of physicians. Accordingly, the complaint with the lowest total SoR score is the most important (Table 2). Thirdly, a 3-point priority card was used. This card ranks the complaints horizontally, based on the physicians' choices in the first, second, and third order. The complaints preferred by the highest number of physicians in the first order are labeled as “Primaries,” followed by “Secondaries” for the second order, and “Tertiaries” for the third order. The 3-point priority card presents the ranking of complaints descriptively and does not involve any summation calculations (Fig. 1).

**Table 1:** Distribution of the ranking of physician complaints.

Rank	A	B	C	D	E	F	G	H	I	J	K	L
**1**	85	33	34	15	43	14	41	52	5	3	74	3
**2**	78	56	40	17	26	30	67	29	6	7	41	4
**3**	45	36	29	3	65	35	39	41	37	18	38	16
**4**	20	53	33	20	44	33	42	54	22	14	37	28
**5**	30	44	47	33	49	31	38	26	19	16	39	27
**6**	43	50	37	11	37	55	28	32	24	26	40	15
**7**	36	31	55	47	15	25	52	45	39	25	10	17
**8**	3	35	38	57	47	35	18	60	37	27	14	24
**9**	12	19	47	60	37	41	40	19	61	19	25	14
**10**	11	14	14	56	19	55	28	19	62	88	12	16
**11**	25	21	12	28	9	30	0	13	50	97	58	50
**12**	11	4	12	49	8	11	7	6	34	56	12	183

N: 402; A: Financial limitations; B: Examination time; C: Length of work hours; D: Shift work; E: Fear of malpractice; F: Mobbing and organizational problems; G: Violence; H: Concerns about the future; I: Mandatory service and appointment barriers; J: Physical conditions; K: Discrediting; L: Problems with legislation and regulations.

**Table 2:** Ranking matrix of important issues for physicians.

Rank (R)	A (f*R)	B (f*R)	C (f*R)	D (f*R)	E (f*R)	F (f*R)	G (f*R)	H (f*R)	I (f*R)	J (f*R)	K (f*R)	L (f*R)
**1**	85	33	34	15	43	14	41	52	5	3	74	3
**2**	156	112	80	34	52	60	134	58	12	14	82	8
**3**	135	108	87	9	195	105	117	123	111	54	114	48
**4**	80	212	132	80	176	132	168	216	88	56	148	112
**5**	150	220	235	165	245	155	190	130	95	80	195	135
**6**	258	300	222	66	222	330	168	192	144	156	240	90
**7**	252	217	385	329	105	175	364	315	273	175	70	119
**8**	24	280	304	456	376	280	144	480	296	216	112	192
**9**	108	171	423	540	333	369	360	171	549	171	225	126
**10**	110	140	140	560	190	550	280	190	620	880	120	160
**11**	275	231	132	308	99	330	0	143	550	1067	638	550
**12**	132	48	144	588	96	132	84	72	408	672	144	2196
**SoR**	**1765**	**2072**	**2318**	**3150**	**2132**	**2632**	**2050**	**2142**	**3151**	**3544**	**2162**	**3739**

A: Financial limitations; B: Examination time; C: Length of work hours; D: Shift work; E: Fear of malpractice; F: Mobbing and organizational problems; G: Violence; H: Concerns about the future; I: Mandatory service and appointment barriers; J: Physical conditions; K: Discrediting; L: Problems with legislation and regulations.

Ishizaka and Lusti [44] presented a comparable technique in the Analytic Hierarchy Process, focusing on column totals. In the healthcare domain, Nobre et al. [41] applied a similar approach for prioritization in multi-criteria decision-making methods. The mathematical formulation of the SoR model is as follows.

**Table:** 

SoR: $\sum_{i=1}^{n}\left(C\left(f\right)\;x\;Ri\right)$; inLogic: $SoR\Rightarrow IR\equiv \left(SoR↑\Lambda \;CR↑\right){⊻(SoR}^{\prime }↑\Lambda \;IR↑)$
**C:***Code*; **Ri:***Rank*; **CR:***Rank of Code*; **Code(f):***The frequency of the observation marking the code*; **n:** Item number; **IR:***Importance in Ranks*

The arrangement of codes from most important to least important, left to right, is achieved using the Importance Rank (IR) in both the SoR model and the 3-point priority card. For example, in a sequence like “AGBEHKCFDIJL,” “A" represents the most important complaint, while “L" represents the least important.

In the third part of the analysis, hypothesis tests were performed on the complaint ranks. The 1st place corresponds to the most important complaint, and the 12th place corresponds to the least important. Therefore, if the ranks are lower in certain groups of variables, it indicates a higher level of importance for that particular group. Non-parametric hypothesis tests were employed since the calculations were based on ranks. The Mann-Whitney *U* test was used for comparisons involving two-group variables, the Kruskal-Wallis H test was used for comparisons involving three or more groups, and the Dunn's test was applied for post-hoc analyses. Spearman's rho correlation coefficient was used to identify relationships among numerical variables. All evaluations were conducted at a 95% confidence level.

### 3.8 Ethical aspect of the research

Ethical approval for the study was obtained from the Istanbul Okan University Ethics Committee following Helsinki Declaration, on August 24, 2022, with decision number 157/16.

### 3.9 Inclusion and exclusion criteria

This research focuses on doctors in Turkey and the inclusion and exclusion criteria are outlined as follows.

#### 3.9.1 Inclusion criteria

• The participant must be an active medical doctor working in a hospital in Turkey.
• The participant should possess adequate professional experience to evaluate the specific difficulties and complaints encountered by doctors, typically requiring a minimum of one year of experience.
• The participant should have the capacity and willingness to complete all sections of the questionnaire.
• The participant must provide signed informed consent for the use of their data.

#### 3.9.2 Exclusion criteria

• The participant is not a medical doctor working in a hospital or is not actively involved in patient care.
• The participant has less than one year of professional experience, making it difficult to fully assess the specific difficulties and complaints faced by doctors.
• The participant faces challenges in responding to the questionnaire or lacks sufficient time to complete it in its entirety.
• The participant declines to sign the informed consent form or withdraws consent during participation.
• The participant expresses any objections to the use of their data after survey completion.

## 4 Results

There were 402 participants, with 227 (56.5%) identifying as female and 175 (43.5%) as male. Among them, 202 (50.2%) were single, while 200 (49.8%) were married. Regarding their professional roles, 40 (10%) were general practitioners, 188 (46.8%) were research assistant doctors undergoing specialization training, and 174 (43.3%) were specialists or doctors holding academic positions such as Assistant Prof., Associate Prof., or Prof. The participants' average age was 34.00 ± 8.84 (ranging from 23 to 65), and they had an average professional experience of 9.39 ± 9.03 years (ranging from 1 to 42).

Table 1 displays the distribution of physicians who ranked complaints in order of importance. Among those who ranked it as the first priority, 85 individuals cited financial limitations, 74 individuals pointed out discrediting, 52 individuals expressed worries about the future, 43 individuals were concerned about fear of malpractice, 41 individuals highlighted violence, 34 individuals mentioned long working hours, 33 individuals brought up examination time, 15 individuals referred to shift working hours, 14 individuals identified mobbing and organizational problems, 5 individuals mentioned mandatory service and assignment barriers, and 3 individuals each indicated inadequate diagnosis and examination due to physical conditions, and legislative and regulatory problems.

Table 2 presents a matrix derived from multiplying the number of physicians who ranked physician complaints in a specific order by the total SoR score. Financial limitations obtained the lowest SoR score (1765), indicating it as the most important physician complaint. Conversely, problems with legislation and regulations obtained the highest SoR score, signifying it as the least important physician complaint. Consequently, the IR ranking was determined as AGBEHKCFDIJL (Fig. 1).

According to the rankings of doctors' complaints, the coding for the 3-point priority card, ranging from most important to least important, was established as follows: “Primary” issues were coded as AKHEGCBDFIJL, “Secondary” issues were coded as AGBKCFHEDJIL, and “Tertiary” issues were coded as EAHGKIBFCJLD (Fig. 1).

Age groups showed significant differences in the importance of working hours, fear of malpractice, mobbing and organizational problems, and mandatory service and appointment barriers. Those under 35 years old placed more importance on working hours and mandatory service and appointment barriers, while those over 35 years old considered malpractice fears, mobbing, organizational problems, and legal issues more significant.

Years of work experience also showed significant differences in the importance of examination time, and mobbing and organizational problems. Those with less than 5 years and more than 15 years of experience considered examination time less important than those with 5–15 years of experience. Mobbing and organizational problems were considered less important by those with 5–15 years of experience compared to other groups. Moreover, those with less than 5 years of experience considered mobbing and organizational problems less important than those with over 15 years of experience.

Marital status showed significant differences in the importance of malpractice fears and discrediting, with single individuals considering malpractice fears less important, and married individuals considering discrediting less important.

Degree-based differences were observed in all problem options. General practitioners considered financial limitations, examination time, shift work, mandatory service and appointment barriers, and physical conditions less important than other doctors. Research assistants and doctors with academic titles (assistant professors, associate professors, and professors) considered violence, future concerns, discrediting, and legal issues less important than general practitioners. General practitioners and doctors with academic titles considered working hours less important than resident assistants. Research assistants considered fear of malpractice and mobbing and organizational problems less important than general practitioners and doctors with academic titles.

The desire to continue practicing medicine in Turkey was more significantly affected by factors such as mobbing and organizational problems than geographical mobility and working conditions abroad (Table 3).

**Table 3:** Comparison of the complaints rankings of physicians according to their characteristics.

	n	A	B	C	D	E	F	G	H	I	J	K	L
		M (Q_1_-Q_3_)	M (Q_1_-Q_3_)	M (Q_1_-Q_3_)	M (Q_1_-Q_3_)	M (Q_1_-Q_3_)	M (Q_1_-Q_3_)	M (Q_1_-Q_3_)	M (Q_1_-Q_3_)	M (Q_1_-Q_3_)	M (Q_1_-Q_3_)	M (Q_1_-Q_3_)	M (Q_1_-Q_3_)
Sex													
Female	227	4 (2–7)	5 (3–7)	6 (4–8)	8 (7–10)	5 (3–8)	6 (4–10)	5 (2–9)	5 (3–8)	9 (5–11)	10 (8–11)	4 (2–8)	11 (6–12)
Male	175	3 (2–6)	4 (2–8)	5 (3–8)	8 (5–10)	5 (4–8)	7 (5–9)	5 (3–7)	5 (3–8)	8 (7–10)	10 (7–11)	5 (2–9)	11 (8–12)
*z*		*−1.122*	*−1.628*	*−1.545*	***−2.004***	*−3.344*	*−.795*	*−.352*	*−.519*	***−2.187***	*−.747*	*−.623*	*−1.387*
*p*		*.262*	*.104*	*.122*	.045*	*.001*	*.426*	*.725*	*.604*	.029*	*.455*	*.533*	*.166*
**Age groups**													
35 years and under	270	3 (2–7)	5 (3–7.25)	5 (3–7.25)	9 (7–10)	5 (3–8)	7 (5–10)	5 (2–7)	5 (2.75–8)	8 (5–10)	10 (7–11)	5 (2–8)	11 (8–12)
Over 35 years	132	3 (1–6)	5 (3–7)	7 (4–9)	8 (5–10)	5 (3–6.75)	6 (4–9)	5 (2–7)	6 (3–8)	9 (7–11)	10 (7.25–11)	6 (2–9)	11 (5–12)
*z*		*−1.871*	*−1.220*	***−4.074***	*−.780*	***−2.215***	***−2.185***	*−.868*	*−1.735*	***−3.637***	*−1.154*	*−1.602*	***−2.206***
*p*		*.061*	*.222*	<.001***	*.436*	.027*	.029*	*.385*	*.083*	<.001***	*.248*	*.109*	.027*
**Years of employment**													
Under 5 years_a_	186	5 (2–7)	6 (3.75–8)	5 (2–8)	9 (7–10)	5 (3–8)	7 (3–10)	5 (3–7)	5 (2–8)	8.5 (5–10)	10 (6–11)	5 (2–8)	11 (8–12)
Between 5 and 15 years_b_	113	3 (2–6)	4 (3–5)	6 (4–7)	8 (6–11)	5 (3–7.5)	8 (6–10)	6 (2–7)	6 (3–8)	8 (5–9)	10 (9–11)	5 (2–10)	12 (8–12)
Above 15 years_c_	103	3 (1–6)	5 (3–8)	7 (6–9)	8 (5–10)	5 (2–8)	5 (3–9)	5 (3–7)	5 (3–8)	10 (7–11)	10 (8–11)	5 (2–9)	10 (5–12)
***χ2***		*1.185*	***17.016***	*1.334*	*.169*	*1.120*	***4.985***	*.407*	*.670*	*1.897*	*.716*	*.027*	*2.678*
*p*		*.276*	<.001***	*.248*	*.681*	*.290*	.026*	*.524*	*.413*	*.168*	*.398*	*.870*	*.102*
*Post-hoc*		–	a,c > b	–	–	–	b>a,c a>c	–	–	–	–	–	–
**Marital Status**													
Single	202	4 (2–7)	5 (2–7)	5 (3–8)	9 (7–10)	5 (3–8)	7 (4–10)	5 (3–7)	5.5 (3–8)	9 (5–10)	10 (6–11)	4 (1–7.25)	11 (7–12)
Married	200	3 (2–6)	5 (3–7)	6 (4–9)	8 (6–10)	4 (3–7)	6 (4–9)	4 (2–7)	5 (3–8)	9 (7–10)	10 (8–11)	5 (3–10)	11.5 (7–12)
*z*		*−1.501*	*−.450*	*−1.657*	*−1.317*	***−2.662***	*−.969*	*−1.360*	*−.567*	*−.746*	*−.770*	***−3.958***	*−1.501*
*p*		*.133*	*.653*	*.097*	*.188*	<0.01**	*.332*	*.174*	*.570*	*.455*	*.441*	<.001***	*.133*
**Degree**													
General practitioner_a_	40	6 (3–6)	7 (4–9)	7 (5–8)	9 (9–10)	4 (3–6)	7 (3–8)	2 (2–5.25)	4 (1–6)	11 (8–11)	12 (10–12)	2.5 (2–4.75)	6.5 (4–11)
Res. Assist._b_	188	3 (2–7)	5.5 (3–7)	5 (2–7)	8 (7–9)	6 (3–9)	7 (5–10)	5 (3–7)	5 (3–8)	8 (4–10)	10 (6–11)	5 (2–9)	12 (9–12)
Specialist or those with academic titles	174	3 (1–6)	4 (3–7)	6 (4–9)	8 (5–11)	5 (2.75–7)	6 (4–9)	5 (2–8)	6 (3–8)	9 (6.75–10)	10 (8–11)	5 (3–9)	11 (7–12)
***χ2***		***8.297***	***15.938***	***27.379***	***8.959***	***20.540***	***6.169***	***18.485***	***6.837***	***25.516***	***27.446***	***12.369***	***18.514***
*p*		.016*	<.001***	<.001***	.011*	<0.001***	.046*	<.001***	.033*	<.001***	<.001***	<0.01**	<.001***
*Post-hoc*		*a>b,c*	*a>b,c*	*a,c > b*	*a>b,c*	*b>a,c*	*b>a*	*b,c>a*	*b,c>a*	*a>b,c;* *c > b*	*a>b,c*	*b,c>a*	*b,c>a*
***Desire to stay in Turkey***													
VAS Type Score		3 (2–6)	5 (3–7)	6 (3–8)	8 (6,75–10)	5 (3–8)	7 (4–9)	5 (2–7)	5 (3–8)	9 (6–10)	10 (7–11)	5 (2–9)	11 (7–12)
*x* ± *s*		*4,42* ± *3,25*	*5,25* ± *2,91*	*5,84* ± *2,96*	*7,96* ± *2,94*	*5,36* ± *2,96*	*6,69* ± *3,05*	*5,14* ± *2,98*	*5,47* ± *3,02*	*8,01* ± *2,91*	*9,00* ± *2,79*	*5,39* ± *3,61*	*9,48* ± *3,19*
*r*		*−.078*	*.025*	*.002*	*−.010*	*.031*	***.106***	***.176***	***−.112***	***−.106***	***−.108***	*.022*	*.053*
*p*		*.118*	*.623*	*.974*	*.835*	*.539*	.034*	<.001***	.024*	.034*	.030*	*.658*	*.285*

*:p < 0.05; **:p < 0.01; ***:p < 0.001; z: *U* test statistics value; χ2: H test chi-square value; r: spearman's rho two tailed; M: Median; Q_1_: 25.percentile; Q_3_: 75.percentile; *x*: Mean; s: Standard Deviation; Post-hoc: Dunn test.

Significant differences were observed in the importance of shift work, and mandatory service and appointment barriers between sexes, with these issues being more significant for men than women.

## 5 Discussion

Our study presents an exploration into the complaints of doctors, focusing on the challenges they face in their medical practice, with the goal of identifying the most common and pressing issues. By examining how these problems vary across different demographic groups, we hope to shed light on the key areas that policymakers should prioritize. This research, conducted with 402 practicing doctors from various regions in Turkey, seeks to contribute to our understanding of the factors influencing doctor migration by ranking their top three complaints from a list of 12 complaint categories. As of August 2022, it is known that 1402 doctors have migrated to different countries, and this number is expected to reach 3000 by the end of 2022 [45]. Clearly, comprehending and addressing the challenges faced by physicians is crucial for improving the quality of healthcare delivery. We hypothesize that by identifying and prioritizing these issues among different demographic and professional groups, we can gain a nuanced understanding of these complex dynamics. Such insights can be valuable for healthcare administrators, enabling them to develop strategies that enhance physician satisfaction and ultimately contribute to better patient care [46]. Moreover, considering the national economy, Turkey has experienced a growth in health tourism, thanks to its well-trained medical professionals [47]. However, it is important to acknowledge a potential risk: an increasing number of physicians contemplating emigration for practice in foreign nations, which may not be fully captured in official statistics. Alongside this trend, there is a growing concern about unaddressed physician grievances. This presents a precarious situation that demands immediate attention and remedial action [47,48].

In our study, based on the responses of participating physicians, we have identified the top three complaints and calculated their priority using the SoR score. The highest-ranked complaints, in order, are financial difficulties, violence, and examination time. Similar concerns about wages and migration have been observed in Poland, where the loss of physicians and nurses to Western countries has been a significant issue, with expectations of increased migration in neighboring countries over time [49]. A study in Pakistan found that low salary profiles among final-year medical students were the primary driver for their intention to migrate [50]. Conversely, an Australian study examining physician preferences for the private and public sectors highlighted factors beyond income, such as clinical and career uncertainty, and flexibility in working hours. This is attributed to medicine being the highest-paying profession in Australia [51]. Humphries et al. [52] noted a peak in physician migration from Ireland to Australia between 2011 and 2013 during an economic stagnation period, followed by a decrease between 2014 and 2018 as economic conditions improved. The increase in physician migration in Turkey, coinciding with the economic crisis in recent years, aligns with our findings on the most significant complaint being financial problems [52,53]. If policymakers fail to address economic challenges and the erosion of physician salaries in the face of inflation, access to healthcare may be significantly at risk, leading to longer waiting times and a scarcity of specialist physicians [49,52,53].

In addition to economic concerns, our study reveals that incidents of violence, which rank as the second greatest concern for physicians, contribute to the risk of physicians leaving their home countries. A study examining the reasons for doctors' emigration in South Africa identified various push factors, including inadequate remuneration considering working conditions, high crime, and violence rates, political instability affecting future expectations, the prevalence of HIV/AIDS, and the decline of education systems [54]. Our study finds that financial difficulties and violence hold the top two positions, emphasizing the global influence of these issues on physician migration and the need for countries to address them accordingly. Another study in Africa highlighted insecurity and instability, alongside educational factors, as significant drivers of doctor emigration [38]. A 2018 survey involving doctors and nurses reported that 46.6% of participants experienced non-verbal forms of violence [55]. A separate study conducted in Turkey, revealed that 6% of patients admitted to committing acts of violence against healthcare workers [56]. Particularly in Asian countries, young doctors and those working in departments such as emergency services, psychiatry, or intensive care units are at a higher risk of encountering violence, affecting nearly half of all doctors [57]. In Pakistan, insufficient security measures have led to doctors losing their lives in violent incidents each year, prompting healthcare workers to leave the country [58]. These studies indicate that incidents of violence significantly contribute to the migration of healthcare personnel to Western countries, including Europe and the US. Therefore, it is crucial to address and mitigate instances of violence to ensure the personal safety of doctors and the uninterrupted provision of healthcare services. Such measures can not only reduce the inclination of doctors to emigrate but also improve the quality and accessibility of healthcare services.

In Turkey, our study reveals that economic concerns and violence serve as triggers for physician emigration. However, our research identifies consultation times as the third major issue among participating doctors. The scarcity of consultation times not only damages the patient-doctor relationship but also stems from a shortage of doctors. A cohort study investigating malpractice cases between 2007 and 2016 highlighted patient assessment, treatment method selection, and insufficient communication among care providers as the primary reasons for malpractice incidents [59]. Another study also emphasized the role of communication deficiencies in malpractice situations [60]. The prominence of consultation times as the third most significant problem for doctors in our research indicates the high risks physicians face in service delivery and the potential compromise of patient health due to inadequate time for proper healthcare provision. The inclusion of malpractice and future concerns as the fourth most critical issues, which also make the top-three priority list, further substantiates this situation. Given that the shortage of consultation times primarily arises from a lack of doctors per capita, it is paradoxical to see physician migration potentially exacerbating the issue. Additionally, a study found that defensive medical practices resulting from the fear of malpractice increased patient costs by 8–20% [61]. Consequently, patients may face the dual risks of receiving suboptimal healthcare and incurring higher healthcare expenses, leading to a decline in the level of healthcare access. Based on these evaluations, it is essential to increase the number of physicians while extending consultation times simultaneously to enhance healthcare services and potentially reduce physician migration.

In our study, several demographic factors were found to exhibit distinct characteristics in the ranking of doctors' complaints. Young doctors under the age of 35 in Turkey, who often undergo specialist training, tend to have more on-call hours, making excessive work hours more significant for them. Conversely, individuals over the age of 35 may consider issues such as fear of malpractice and mobbing more crucial due to their extensive experience. Moreover, the likelihood of being married is higher in this age group, potentially increasing the importance of malpractice risk due to added responsibilities. For example, a study conducted in Romania identified gender, working more shifts, and being married to a spouse in the same specialty as distinctive factors influencing physicians' concerns regarding malpractice complaints [12].

In Turkey, doctors undergoing specialist training are referred to as “Research Assistants.” These research assistant physicians, as well as specialist doctors or doctors with academic positions, appear to be more troubled by financial matters compared to general practitioners. In contrast to our study, research conducted in the United States compared the compensation provided to primary healthcare providers with that given to specialist doctors, revealing significant pay disparities between doctors working in primary healthcare [62]. However, in Turkey, the compensation gap between doctors working in primary healthcare and specialist doctors is relatively minor. It is also important to consider that physicians preparing for specialization examinations, which involve 3–7 years of education depending on the specialty field, may have higher financial expectations. Furthermore, since research assistant physicians are doctors pursuing specialization, their expectations within this group align closely with those of specialist doctors, supporting this interpretation.

Our research also revealed a noteworthy finding: there is a positive correlation between the desire to remain in Turkey and the underestimation of issues such as mobbing, organizational problems, and violence against doctors among the participating physicians. Conversely, these two concerns can be interpreted as factors that support doctor migration. Moreover, doctors who assign less importance to mobbing, organizational issues, and violence may have come to accept the challenges and limitations inherent in medical practice in Turkey, which could influence their decision to stay. However, a study has indicated that workplace violence and mobbing significantly reduce doctors' job satisfaction [13]. Additionally, it is crucial to address preventable issues like violence and malpractice promptly as the initial step. These factors are integral to healthcare, considering the demanding nature of work environments, high workloads, and lengthy and costly training processes.

## 6 Conclusion

We explored the primary grievances contributing to the growing trend of doctor emigration in Turkey. Alongside significant concerns such as financial challenges, incidents of violence, and limited consultation time, we also observed complaints related to mobbing, organizational issues, malpractice, and anxieties about the future. The ranking of doctors' complaints can vary depending on demographic factors and professional roles, providing valuable insights into their expectations when viewed from the opposite perspective.

Considering the limited research available on doctor migration in the literature, we believe that our study, which prioritizes the complaints driving physician emigration in Turkey, can offer valuable insights to countries facing similar challenges. Based on the identified grievances, we propose the following recommendations.

• Policymakers should develop strategies to alleviate the financial burdens faced by doctors. This could involve revising compensation structures in light of the current economic scenario and improving working conditions.
• The implementation of stronger measures against workplace violence should be considered. This could include stricter legislation, the enforcement of penalties, and the promotion of awareness and prevention programs.
• Efforts should be made to increase the number of doctors to provide them with more consultation time and alleviate their pressures. This can be achieved through incentivized medical education programs or recruitment initiatives targeting doctors overseas.
• Support mechanisms to address mobbing and organizational issues can also be established. This could involve creating employee assistance programs, setting up effective grievance resolution mechanisms, and promoting a positive organizational culture.

## 7 Limitations

The study has certain limitations related to its design, data collection approach, and the interpretation of statistical methods used. As a cross-sectional study, it provides a snapshot of the situation at a specific moment, potentially missing out on temporal shifts and evolving trends. The study focuses exclusively on doctors in public hospitals, neglecting potential issues among doctors in private practice or non-hospital settings. Geographically, although the study covers seven Turkish cities and aims to capture various demographic and geographical contexts, it may not fully represent all regions of the country. There might also be underrepresentation of differences between urban and rural areas due to insufficient sample representation. While the sample size of 402 doctors was statistically determined, it constitutes a small fraction of all doctors working in Turkey, which may limit the generalizability of the results. The use of a multi-stage cluster sampling technique could introduce selection bias. The reliance on self-reported data is subject to various factors, including recall bias, social desirability bias, and subjective perceptions of complaint importance. Despite expert reviews and pilot testing, the survey design may not have captured all possible complaints that doctors could have. The predetermined complaint categories might have hindered the expression of unique or less frequent complaints. The use of non-parametric tests for demographic comparisons limits the adjustment for confounding variables, requiring careful interpretation of demographic differences. Despite these limitations, the study provides valuable insights into the complaints and concerns of doctors in public hospitals in Turkey, which can inform future interventions to improve working conditions and job satisfaction. Future research should consider longitudinal designs to capture temporal changes, larger and more diverse samples across different regions, and comprehensive data collection methods to address the mentioned limitations.

## Author contribution statement

Salim Yılmaz, Ph. D: Conceived and designed the experiments; Performed the experiments; Analyzed and interpreted the data; Contributed reagents, materials, analysis tools or data; Wrote the paper.

Seher Koyuncu Aydın, MD: Conceived and designed the experiments; Performed the experiments.

## Data availability statement

Data will be made available on request.

## Declaration of competing interest

The authors declare that they have no known competing financial interests or personal relationships that could have appeared to influence the work reported in this paper.
